# Very Long‐Term Follow‐Up of Multidimensional Health‐Related Quality of Life After Endoscopic Endonasal Surgery for Pituitary Adenomas: A Prospective Cohort Study

**DOI:** 10.1002/hed.70103

**Published:** 2025-11-28

**Authors:** Gonneke E. Joustra, Nathalie F. van Rhee, Marc C. den Heijer, Astrid G. W. Korsten‐Meijer, Robert A. Feijen, György B. Halmos, Jos M. A. Kuijlen, Karin M. Vermeulen

**Affiliations:** ^1^ Department of Otorhinolaryngology—Head and Neck Surgery, University Medical Center Groningen University of Groningen Groningen the Netherlands; ^2^ Graduate School of Medical Sciences University of Groningen Groningen the Netherlands; ^3^ Department of Neurosurgery, University Medical Center Groningen University of Groningen Groningen the Netherlands; ^4^ Department of Epidemiology, University Medical Center Groningen University of Groningen Groningen the Netherlands

**Keywords:** anterior skull base, Endoscopic Endonasal Sinus and Skull Base Surgery Questionnaire (EES‐Q), endoscopic skull base surgery, patient‐reported outcome measure, pituitary adenoma

## Abstract

**Background:**

To evaluate postoperative health‐related quality of life (HRQoL) for pituitary adenoma patients, multidimensional assessment is important. Available data is limited on long‐term follow‐up.

**Methods:**

Prospectively, 52 nonfunctioning (NFA) and functioning (FA) pituitary adenoma patients were included. The Endoscopic Endonasal Sinus and Skull Base Surgery Questionnaire (EES‐Q) was completed preoperatively and postoperatively (2 weeks, 3 months, 1 year, 8.5 years). Generalized estimating equations identified variables associated with HRQoL changes over time.

**Results:**

Psychological (*p* = 0.006) and social HRQoL (*p* = 0.026) significantly improved after very long‐term follow‐up, while physical HRQoL and EES‐Q scores normalized. Pre‐ and postoperatively, most inconveniences were reported in the social domain, with a noticeable difference between NFA and FA patients. Female gender negatively impacted physical HRQoL.

**Conclusions:**

Endoscopic endonasal surgery has no very long‐term negative impact on HRQoL. Social functioning remained the most affected throughout follow‐up. These results highlight the importance of this multidimensional HRQoL tool to improve patient‐centered health care.

## 1 Introduction

Pituitary adenomas, tumors originating in the sellar region of the anterior skull base, represent one of the most prevalent types of intracranial tumors [1]. Classified by size and functional status, patients typically present with hormonal dysregulation or mass‐induced compressions on surrounding structures such as the optic chiasm [2]. Pituitary adenomas can be safely and effectively treated with endoscopic endonasal surgery (EES) [3].

Today, health‐related quality of life (HRQoL) is an important endpoint in medical treatment and requires a patient‐centered approach [4, 5, 6]. Assessment of HRQoL should be multidimensional and include a physical, psychological, and social domain [7, 8]. Frequently used disease‐specific questionnaires do not capture all three HRQoL domains, do not assess nasal morbidity or are not validated for EES or pituitary adenomas [9, 10].

The Endoscopic Endonasal Skull Base Surgery Questionnaire (EES‐Q, Appendix A) was previously developed and validated by our research group [9, 11]. The EES‐Q is a multidimensional, reliable and comprehensive tool suitable for daily practice and scientific research, fully aligned with the HRQoL concept [6, 9, 12].

Our research group previously evaluated HRQoL in pituitary adenomas up to 1 year following EES [12]. Few studies assessed HRQoL beyond 5 years postoperatively [4, 13, 14, 15]. Recent prospective studies use multiple HRQoL questionnaires to evaluate different health domains, in contrast to the comprehensive multidimensional EES‐Q [16, 17, 18]. In addition, these studies lack very long‐term follow‐up and are limited by small sample sizes [16, 17, 18]. Furthermore, to the best of our knowledge, there is no internationally accepted gold standard for rhinological follow‐up after endonasal surgery for pituitary adenomas. In the Netherlands, current guidelines recommend preoperative evaluation by a rhinologist and postoperative consultation as required [19].

This prospective study evaluates the very long‐term impact of EES on multidimensional HRQoL in patients with pituitary adenomas, including both functioning adenoma (FA) and nonfunctioning adenoma (NFA), using the EES‐Q. Furthermore, factors influencing the postoperative HRQoL were identified. The findings enhance patient‐centered (preoperative) counseling and contribute to the development of evidence‐based postoperative follow‐up recommendations.

## 2 Materials and Methods

This prospective study was performed in a tertiary referral center at the Departments of Otorhinolaryngology—Head and Neck Surgery and Neurosurgery. Institutional review board approval was obtained before commencing.

### 2.1 Participants

Since this was a very long‐term follow‐up within an existing cohort, no sample size calculation was performed. We invited all available patients to participate between August and December 2023. A total of 52 of the 101 patients who had previously been enrolled in the 1‐year follow‐up study were included in this study. The remaining 49 patients were lost to follow‐up: incorrect contact details (15, 30.6%), deceased (10, 20.4%), or no longer interested in participating (24, 49.0%). The inclusion criteria were (1) aged ≥ 18 years; (2) able to read and write Dutch; and (3) treated by EES.

### 2.2 Study Design

Demographic data and EES‐Q results of our previously included patients were analyzed. For the very long‐term follow‐up patients were contacted and written informed consent was obtained. The participants then received the EES‐Q digitally. In total, the EES‐Q was completed 1 day preoperatively and postoperatively (2 weeks, 3 months, 1 year, very long‐term).

### 2.3 The Endoscopic Endonasal Sinus and Skull Base Surgery Questionaire (EES‐Q)

The EES‐Q is a validated, patient‐reported outcome questionnaire (30 items) in a physical, psychological, and social domain (Appendix A) [11]. A 5‐point Likert response scale, ranging from *not at all* (1) to *very severely* (5) is used to indicate the degree of inconvenience. Lower scores indicate better HRQoL. Completion typically requires 3–5 min. *Domain scores* were calculated to create an easily interpretable score, ranging from 0 (not bothered at all) to 100 (very severely bothered). *Domain scores* were calculated by summing the 10‐item score of each domain, subtracting 10 points from this total and multiplying this by 2.5. The *EES‐Q score* is created by summing all individual scores of the 30 questions and dividing this by 3, indicating equal importance of all domains.

### 2.4 Surgical Technique

All patients underwent a similar endoscopic transsphenoidal approach performed by a surgical team consisting of a rhinologist and a neurosurgeon [3, 20]. Only in rare cases was the middle turbinate (partly) resected with or without an ethmoidectomy. After a wide bilateral sphenoidectomy with partial removal of the posterior septum, anatomical landmarks such as the optic carotic recess were identified. A rescue nasoseptal flap (NSF) was prepared. The tumor was removed after drilling the sellar floor and opening the epidural layer. For suprasellar adenomas the tuberculum sellae and/or the planum sphenoidale were opened. After removal of the tumor a multilayer reconstruction (including gasket seal technique) with or without a NSF was performed.

### 2.5 Follow‐Up

Very long‐term routine follow‐up was conducted by an endocrinologist. There is currently no standardized protocol for the postoperative involvement of other specialists, including a rhinologist, neurosurgeon or ophthalmologist. In our center, based on expert opinion, the rhinological follow‐up is routinely conducted 2 weeks and 3 months postoperatively. Patients were considered in ‘remission’ when they had no biochemical (obtained from the endocrinologist's reports), clinical or radiological signs of active disease.

### 2.6 Statistical Analysis

Descriptive statistics were used to summarize patients' demographics. Mean and standard deviation (SD) for all domains were calculated at every follow‐up point to indicate the trend in HRQoL. Appropriate tests were performed (Wilcoxon signed rank test or Mann–Whitney test for skewed data, independent *t* test for normally distributed data). For means of interpretability the mean (SD) is reported instead of the median score. A generalized estimating equation (GEE) (univariate and, if applicable multivariate) analysis was performed on previously selected covariates to identify significant longitudinal changes in HRQoL over time and was reported using the *p* value (Table 1) [12]. A *p*‐value< 0.05 was considered statistically significant. The statistical analyses were performed using IBM Statistics SPSS version 28 (SPSS IBM Inc.).

**TABLE 1:** Covariates.

Covariate		
Gender	Male	Female
Age	≤ 60 years	> 60 years
Postoperative CSF leakage	Yes	No
Prior EES	Yes	No
Parasellar invasion (Knosp)	< Grade III	≥ Grade III
Suprasellar invasion (Hardy–Wilson)	< C	≥ C
Tumor size	< 10 mm	≥ 10 mm
Nonfunctioning adenoma	Yes	No
Endocrine remission within 6 months postoperatively	Yes	No
Adjuvant radiotherapy	Yes	No
Need for reoperation	Yes	No
Body mass index	< 30 kg/m^2^	≥ 30 kg/m^2^

*Note*: Covariates selected to assess their effect on the trend in HRQoL over time are shown [12].

Abbreviations: CSF, cerebrospinal fluid; EES, endoscopic endonasal surgery.

## 3 Results

### 3.1 Patient Characteristics

A total of 52 patients (50% female) were included. The mean age was 61.7 ± 11.3 years. The mean very long‐term follow‐up was 101.7 ± 11.8 months. Thirty‐seven (71.2%) patients were diagnosed with a macroadenoma (mean 25.9 ± 9.8 mm) and 15 patients (28.8%) with a microadenoma (mean 6.1 ± 2.1 mm). Twenty‐nine patients (55.8%) were diagnosed with NFA, and 23 patients (44.2%) with FA (Table 2). In this cohort, reconstruction with a NSF was not performed. Postoperative CSF leak was present in three (5.8%) patients (one microadenoma/FA, two macroadenoma/NFA), all received an external lumbar drain. Two patients (3.8%) (both macroadenoma/NFA) developed persistent diabetes insipidus. No other persistent complications were observed in this cohort.

**TABLE 2:** Patients' characteristics.

Characteristics	*N* (%)
Gender (female)	26 (50)
Mean (SD) age (in years)	61.7 (11.3)
Mean (SD) BMI (kg/m^2^)	29.0 (5.2)
History of EES prior to pituitary EES	8 (15.4)
*Macroadenoma (4 giant adenomas)*	37 (71.2)
NFA	29 (78.4)
Cushing	1 (2.7)
Acromegaly	5 (13.5)
Prolactinoma	2 (5.4)
*Macroadenoma expansion*	
Parasellar invasion (Knosp score ≥ 3)	14 (37.8)
Suprasellar invasion (≥ Hardy–Wilson Grade C)	5 (13.5)
*Microadenoma*	15 (28.8)
Cushing	12 (80.0)
Acromegaly	1 (6.7)
Prolactinoma	1 (6.7)
TSH	1 (6.7)
*Re‐intervention during follow‐up*	22 (42.2)^a^
Surgery	9 (40.9)
Radiotherapy	13 (59.1)
*Remission*	47 (90.4)
With medication (supplementary or suppressive)	32 (68.1)
Without medication	15 (31.9)

Abbreviations: BMI, body mass index; EES, endoscopic endonasal surgery; NFA, nonfunctioning adenoma; SD, standard deviation; TSH, thyroid‐stimulating hormone.

^a^ Four patients required more than one intervention postoperatively.

Twenty‐two (42.2%) patients (18 macroadenoma; 4 microadenoma) required re‐intervention during follow‐up, more than 60% in the first 4 years postoperatively. Thirteen (44.8%) NFA patients had adjuvant surgery (5; 38.5%) or radiotherapy (8; 61.5%). After very long‐term follow‐up most NFA patients were in remission (27; 93.1%) with (21; 72.4%) or without (6; 20.7%) medication. Two NFA patients were scheduled for adjuvant surgery or required medical treatment. Nine (39.1%) FA patients underwent additional therapy: 4 (44.4%) adjuvant surgery, 5 (55.6%) radiotherapy. Most FA patients were in remission (20; 87.0%) with (11; 55.0%) or without (9; 45.0%) medication. Three patients with recurrent biochemical activity were closely monitored, required medical treatment or were scheduled for adjuvant surgery.

### 3.2 Health‐Related Quality of Life

#### 3.2.1 Effect of EES on Very Long‐Term HRQoL

**FIGURE 1:**
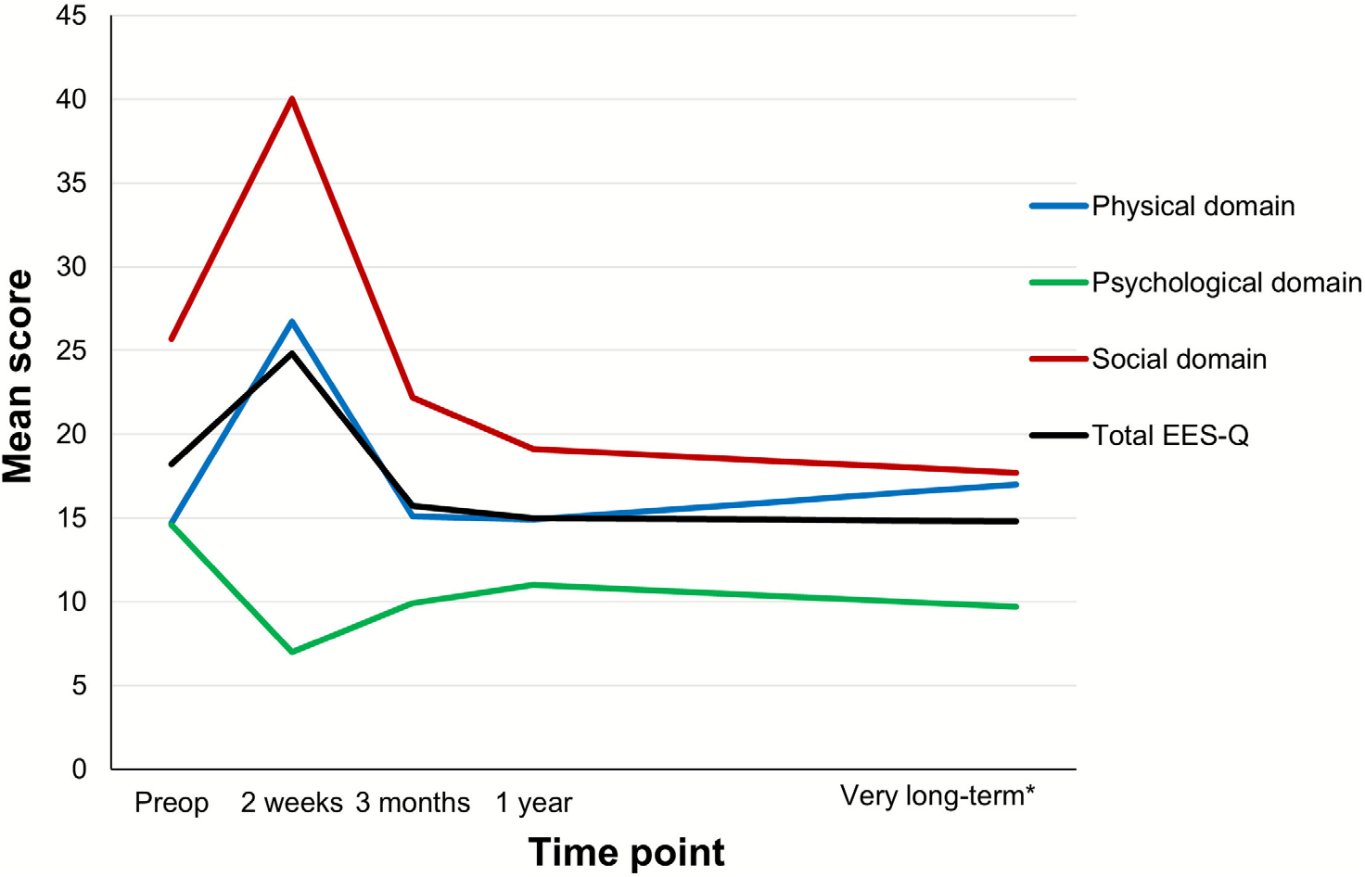
Mean domain and EES‐Q scores—all patients. The mean EES‐Q and domain (physical, psychological, and social) scores preoperatively and postoperatively (2 weeks, 3 months, 1 year, very long‐term) are shown. Lower scores represent better HRQoL. *101.7 ± 11.8 months. [Color figure can be viewed at wileyonlinelibrary.com]

EES‐Q scores were significantly worse 2 weeks postoperatively (*p* = 0.014), compared with preoperatively. However, by 3 months postoperatively no significant difference was observed (*p* = 0.206) and scores remained stable thereafter (Figure 1). Physical HRQoL showed a significant decline 2 weeks postoperatively (*p* < 0.001). From 3 months onwards, no significant differences were noted compared with preoperative scores (*p* = 0.961); this continues during follow‐up (Figure 1). Psychological HRQoL significantly improved 2 weeks postoperatively (*p* < 0.001). Although a slight decline was observed over time, psychological HRQoL remained significantly better than preoperatively at 3 months (*p* = 0.028), 1 year (*p* = 0.011), and very long‐term follow‐up (*p* = 0.006) (Figure 1). Social HRQoL was significantly worse 2 weeks postoperatively (*p* = 0.005). From 3 months onwards, a gradual improvement was observed, although not reaching significance in the first year (*p* = 0.686 and *p* = 0.141, respectively). At the end of the follow‐up period, the social HRQoL was significantly better compared with preoperatively (*p* = 0.026) (Figure 1).

EES‐Q and domain scores did not significantly differ between patients with or without recurrent disease (*p* = 0.860). There was no difference reported between patients with or without medication use (*p* = 0.243).

#### 3.2.2 NFA vs FA Patients

**FIGURE 2:**
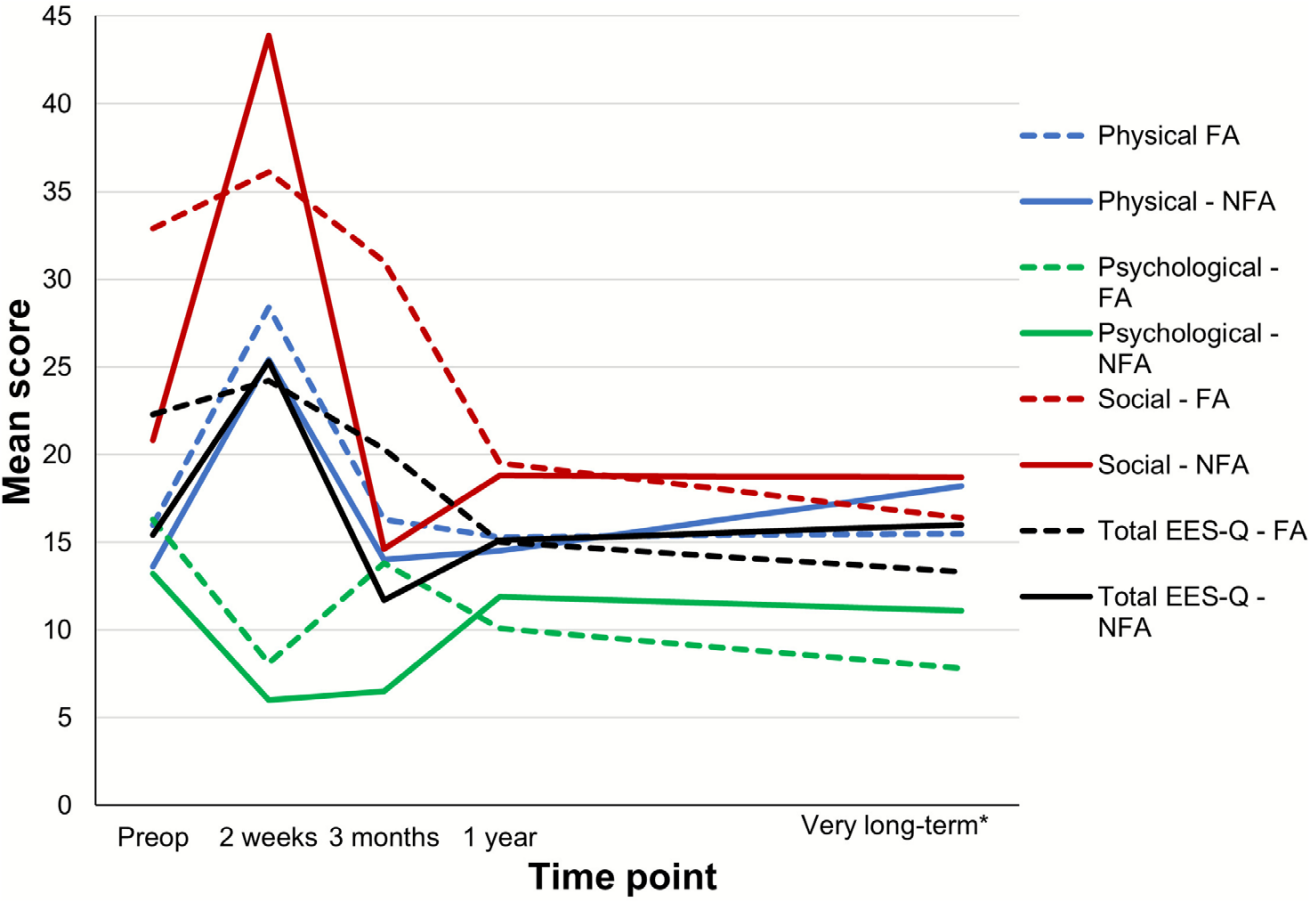
Mean domain and EES‐Q scores—NFA versus FA. The mean EES‐Q and domain (physical, psychological, and social) scores preoperatively and postoperatively (2 weeks, 3 months, 1 year, very long‐term) are shown. Lower scores represent better HRQoL. *101.7 ± 11.8 months. [Color figure can be viewed at wileyonlinelibrary.com]

Preoperatively, FA patients reported worse EES‐Q and domain scores compared with NFA patients, although not significant (EES‐Q: *p* = 0.085, physical: *p* = 0.243, physiological: *p* = 0.860, and social domain score: *p* = 0.114) (Figure 2). Very long‐term social HRQoL was significantly improved for FA patients compared with preoperative scores (*p* = 0.002). NFA patients returned to baseline levels and showed no significant improvement compared with preoperatively (*p* = 0.575). Psychological HRQoL in FA patients also improved significantly compared with preoperatively (*p* = 0.002). NFA patients returned to baseline level and showed no psychological improvement (*p* = 0.241) (Figure 2).

#### 3.2.3 Longitudinal Patterns of HRQoL—Univariate Analysis

**FIGURE 3:**
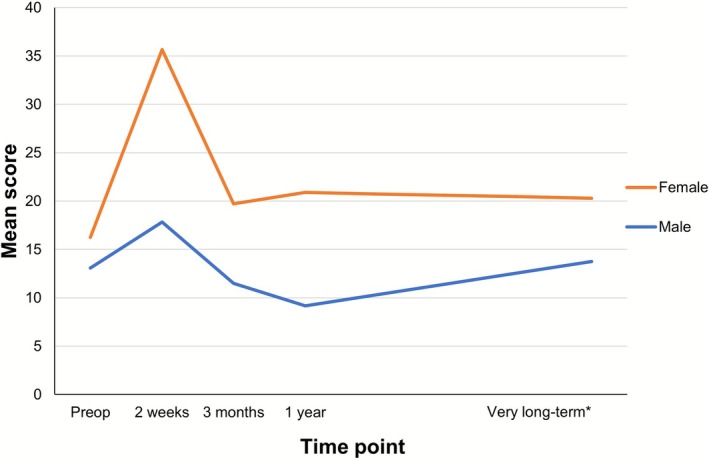
Mean physical domain scores—gender. The mean physical domain scores preoperatively and postoperatively (2 weeks, 3 months, 1 year, very long‐term) are shown. Lower scores represent better HRQoL. *101.7 ± 11.8 months. [Color figure can be viewed at wileyonlinelibrary.com]

Univariately, GEE analysis indicated only a significant difference in physical HRQoL between male and female respondents. The other covariates did not reach significance. Prior to surgery, female patients reported a significantly lower physical HRQoL compared to male patients. Following surgery, there was a marked deterioration in HRQoL for female patients (Figure 3). Two weeks postoperatively males reported a better physical HRQoL compared with females (*β* = −21.10, *p* < 0.001). During very long‐term follow‐up, all patients reported a significant improvement compared with 2 weeks postoperatively (*β* = −5.04, *p* < 0.001). Yet males maintained a better physical HRQoL at each follow‐up point compared with females. Both males and females returned to preoperative scores 3 months postoperatively and remained stable during follow‐up (Figure 3).

## 4 Discussion

### 4.1 Key Findings

This study shows that EES has no negative effects on very long‐term multidimensional HRQoL. After expected postoperative changes, HRQoL normalized or improved from 3 months onwards for all patients. Pre‐ and postoperatively, most inconveniences were reported in the social domain, with a noticeable difference between NFA and FA patients. These results highlight the value of the EES‐Q and contribute to improved pre‐ and postoperative patient counseling. Routine long‐term rhinological follow‐up does not offer significant additional clinical benefit.

### 4.2 Health‐Related Quality of Life

#### 4.2.1 Social Domain

Today, the importance of social HRQoL on overall HRQoL is well‐known [21, 22]. Meaningful social connections and positive social activities are essential contributors to social HRQoL [21, 22]. It is well known that patients with pituitary adenomas have worse HRQoL than healthy people, even after treatment [13, 14, 15, 23]. Nonetheless, social HRQoL in relation to the surgical intervention remains underreported in the literature. Our study highlights the importance of a multidimensional HRQoL questionnaire including the social domain, as patients in our cohort reported the greatest preoperative and postoperative impairments in social HRQoL, compared with the physical and psychological domains. Comparison with existing literature is challenging as the reported results are based on individual items within HRQoL tools, rely on generic HRQoL instruments, or do not specifically evaluate postoperative results. Furthermore, the majority of the studies have shorter follow‐up periods [12, 24, 25, 26]. In line with our results, an improvement in social HRQoL was reported up to 1 year postoperatively [12, 24, 25, 26]. After an initial decline 2 weeks postoperatively, social HRQoL continued to improve during further follow‐up. This trend was expected based on our 1‐year follow‐up results that indicated an improvement in social HRQoL over time [12]. This result is attributable to high remission rates (90.4%) and is consistent with our experience that, over time, a stable disease causes less impact on social health. Even though social HRQoL was worse preoperatively and during follow‐up compared with physical HRQoL it does follow the same trend. This suggests that social functioning may be affected by physical impairments and hormonal imbalances [14, 23, 27, 28, 29]. The significant improvement in social health in our FA population might be attributed to less hormonal imbalance during follow‐up compared with preoperatively [14, 23, 28, 29].

#### 4.2.2 Psychological Domain

Two weeks postoperatively, a significant improvement in psychological HRQoL was reported, which stabilized after 3 months. This is in line with our 1‐year follow‐up results [12]. A gradual improvement and stabilization between 6 and 12 months has previously been reported [5, 24, 30]. Pituitary adenoma patients report worse psychological HRQoL compared with healthy individuals, with evidence suggesting that this may be more pronounced in patients with FA [13, 14, 15, 23, 24, 31, 32]. However, very long‐term postoperative effects are unclear. After very long‐term follow‐up of FA patients in our cohort, a significant improvement in psychological health was found compared with preoperatively and NFA patients returned to preoperative levels. The difference between FA and NFA patients might be attributed to improved hormonal imbalances in the FA group postoperatively. Unfortunately, our group size is too small for subgroup analyses.

#### 4.2.3 Physical Domain

As expected, there was an initial worsening of sinonasal complaints postoperatively, with a return to preoperative scores 3 months postoperatively. This is in accordance with previously reported recovery up to 6 months after EES [12, 33, 34]. As can be seen in Figure 2, the physical HRQoL noticeably and significantly declined between 1 year postoperatively and very long‐term follow‐up. The clinical relevance of this is uncertain as overall physical health tends to decline with age and older patients may experience more nasal symptoms [35, 36]. In line with previous studies, no significant difference was found between FA and NFA patients preoperatively and during follow‐up [12, 37, 38].

### 4.3 Longitudinal Patterns of HRQoL

In accordance with previous studies, female gender negatively impacted physical HRQoL [12, 13, 24, 26, 28, 34]. In our study, males reported a better physical HRQoL preoperatively and during follow‐up compared with females. Even though, no clear explanation can be formulated, it is speculated that differences in sexual hormones and emotion regulation may influence HRQoL [39, 40, 41]. The results within the social domain tended to differ between males and females after initial worsening 2 weeks postoperative, although the results were not significant. Males tended to recover within 3 months, whereas females showed a continuous slow improvement during follow‐up (results not shown). In contrast to our 1‐year follow‐up study, age did not significantly impact physical and social HRQoL [12]. However, the patients > 60 years appeared to physically recover more slowly after EES compared with younger patients (results not shown). Although older age is associated with better coping and emotional regulation, physiological factors of aging may negatively influence physical HRQoL over time [40].

### 4.4 Recurrence and Remission

During follow‐up, 42.2% of patients required adjuvant postoperative therapy, of which 81.8% were diagnosed with a macroadenoma. The results were similar to previous macroadenoma studies with subtotal resection increasing the need for adjuvant therapy [42, 43, 44]. In several studies adjuvant therapy was associated with a worse HRQoL after EES [5, 26, 28, 42] whereas in other studies it was not [13, 45]. Our study did not report any long‐term differences in HRQoL in patients with or without medication use. In line with reported remission rates ranging from 30% to 92% [46, 47, 48] our study reported an overall remission rate of 90.4%. Given the substantial influence of disease type on remission rates, meaningful comparison with other studies was not possible. Postoperative treatment and pituitary hypofunction are known to negatively affect HRQoL [26, 28, 49, 50]. In our study, patients requiring medication reported a remarkable but not significantly worse HRQoL in the psychological domain.

### 4.5 Limitations

The authors acknowledge the relatively small sample size. However, considering the very long‐term follow‐up with a mean of over 100 months the current participation rate was perceived as substantial. The authors feel that the very long‐term results of this study may apply to a larger group of patients since the results are almost identical to our previous 1‐year follow‐up findings in a larger cohort [12]. The number of patients included in the analyses up to 1 year postoperatively varies due to missing data. Since then, there is no missing data as the EES‐Q is digitally available with an automatic control to prevent missing data. The GEE can validly handle missing data. Comparison of health domains with existing literature was challenging due to the use of various HRQoL questionnaires, highlighting the advantage of a single multidimensional HRQoL tool. Even though the EES‐Q is not yet widely adopted, it provides meaningful information on the burden of EES in all three health domains in one instrument; it is suitable for daily practice and scientific research. To gain more insight into the details of the specific subgroups, additional research with larger study populations is advised.

### 4.6 Conclusions

Surgical treatment of pituitary adenomas via an endoscopic endonasal approach has no negative effects on HRQoL. Furthermore, our current approach on rhinological follow‐up proves to be effective and efficient. After expected postoperative changes, HRQoL normalized or improved from 3 months onwards for all patients. The most noteworthy change in HRQoL is evident in social functioning, which remains the most affected domain pre‐ and postoperatively. The EES‐Q provides meaningful information to improve all aspects of patient‐centered health care, and our results highlight the importance of this multidimensional HRQoL tool. We believe, it is imperative to evaluate the (very) long‐term outcomes of any surgical intervention. These results provide a solid foundation for integrating this information into preoperative counseling, thereby enhancing informed decision‐making.

## Author Contributions

All authors contributed to the study conception and design. Material preparation, data collection, and analysis were performed by G.E.J., N.F.R., and K.M.V. The first draft of the manuscript was written by G.E.J. and N.F.R. and all authors commented on previous versions of the manuscript. All authors read and approved the final manuscript.

## Funding

The authors received no specific funding for this work.

## Ethics Statement

Approval by the Medical Ethical Committee of the University Medical Center Groningen was obtained before commencing (METc 2013.251).

## Conflicts of Interest

The authors declare no conflicts of interest.

## Data Availability

The data that support the findings of this study are available from the corresponding author upon reasonable request.

## Appendix A Endoscopic Endonasal Sinus and Skull Base Surgery Questionnaire (EES‐Q)

This questionnaire contains complaints that can be the result of a disorder of the paranasal sinuses. For each complaint, indicate whether you have experienced it *in the past 2 weeks* and if so, how severe it was.

Activities are also mentioned in which you may be hindered in performing as a result of your complaints. For each activity, indicate to what extent you have been *hindered* in the past 2 weeks.

**Table:**

	Not at all	Mildly	Moderately	Severely	Very severely
Blocked nose	□	□	□	□	□
2 Difficulty breathing through nose	□	□	□	□	□
3 Dry mouth	□	□	□	□	□
4 Need to blow nose	□	□	□	□	□
5 Reduced sense of smell	□	□	□	□	□
6 Reduced sense of taste	□	□	□	□	□
7 Facial pressure	□	□	□	□	□
8 Fitful sleep	□	□	□	□	□
9 Tired	□	□	□	□	□
10 Waking up tired	□	□	□	□	□
11 Feeling depressed	□	□	□	□	□
12 Sad	□	□	□	□	□
13 Feeling down	□	□	□	□	□
14 Distressed	□	□	□	□	□
15 Aggravated	□	□	□	□	□
16 Frustrated	□	□	□	□	□
17 Stressed	□	□	□	□	□
18 Tense	□	□	□	□	□
19 Worried	□	□	□	□	□
20 Discouraged	□	□	□	□	□
21 Work/study	□	□	□	□	□
22 Leisure time activities	□	□	□	□	□
23 Hobbies	□	□	□	□	□
24 Indoor activities at home	□	□	□	□	□
25 Outdoor activities at home	□	□	□	□	□
26 Going out for a visit	□	□	□	□	□
27 Shopping	□	□	□	□	□
28 Daily activities	□	□	□	□	□
29 Engaging in outdoor activities	□	□	□	□	□
30 Physical exertion	□	□	□	□	□

*Source*: Ten Dam et al. [11].
